# Weekly gemcitabine plus 24-h infusion of high-dose 5-fluorouracil/leucovorin for locally advanced or metastatic carcinoma of the biliary tract

**DOI:** 10.1038/sj.bjc.6601796

**Published:** 2004-04-13

**Authors:** C Hsu, Y-C Shen, C-H Yang, K-H Yeh, Y-S Lu, C-H Hsu, H-T Liu, C-C Li, J-S Chen, C-Y Wu, A-L Cheng

**Affiliations:** 1 Department of Oncology, National Taiwan University Hospital, 7, Chung-Shan South Road, Taipei 100, Taiwan; 2 Department of Internal Medicine, National Taiwan University Hospital, 7, Chung-Shan South Road, Taipei 100, Taiwan; 3 Department of Internal Medicine, Cathay General Hospital, 280, Jen-Ai Road, Sec 4, Taipei 106, Taiwan; 4 Department of Internal Medicine, Buddhist Tzu-Chi General Hospital, 707, Chung-Yang Road, Hualien 970, Taiwan; 5 Department of Internal Medicine, Chang-Gung Memorial Hospital, 5, Fushing Road, Gueishan Taoyuan 333, Taiwan; 6 Division of Cancer Research, National Health Research Institutes, 128, Yen-Chiu-Yuan Road, Sec 2, Taipei 115, Taiwan

**Keywords:** biliary tract carcinoma, gemcitabine, 5-fluorouracil, leucovorin

## Abstract

Both gemcitabine and weekly 24-h infusion of high-dose 5-fluorouracil/leucovorin (HDFL) have shown promising antitumour activity for patients with locally advanced or metastatic carcinoma of the biliary tract (CBT). From April 1999 through December 2002, 30 patients with inoperable CBT were treated with gemcitabine 800 mg m^−2^, intravenous infusion for 30 min, followed by 5-FU, 2000 mg m^−2^ and leucovorin, 300 mg m^−2^, intravenous infusion for 24 h, on day 1, 8 and 15, every 4 weeks. A total of 166 cycles were given (median of four cycles per patient, range 1–24 cycles). Response was evaluable in 28 patients and toxicity in 29 patients. Partial response was obtained in six patients, stable disease in 13, while progressive disease occurred in nine. The objective response rate was 21.4% (95% CI: 5.2–37.6%). The most common grade 3 or 4 toxicity was infection (nine patients). Other types of grade 3 or 4 toxicity included leucopenia (four patients), thrombocytopenia (three patients), anaemia (three patients), nausea/vomiting (two patients) and elevation of liver transaminases (three patients). As of 30 September 2003, the median progression-free survival was 3.7 months (95% CI: 2.8–4.6 months) and the median overall survival was 4.7 months (95% CI: 0.8–8.6 months). Our data suggest that weekly gemcitabine plus HDFL is modestly active with acceptable treatment-related toxicity for patients with advanced CBT.

Carcinoma of the biliary tract (CBT) is uncommon, accounting for about 5% of primary cancers of the hepatobiliary system. Clinically, CBT is characterized by a very poor prognosis owing to late diagnosis, anatomic limitation for radical resection, early dissemination and lack of effective treatment other than surgery. Most patients with advanced disease die of hepatic failure or biliary sepsis within 6–12 months of diagnosis (de Groen *et al*, 1999).

The results of systemic chemotherapy for CBT have been disappointing. 5-Fluorouracil (5-FU) remains the most effective agent with a tumour response rate of around 10% (Falkson *et al*, 1984; Oberfield and Rossi, 1988). The addition of other agents, such as mitomycin C, doxorubicin or cisplatin, has not shown consistent benefit in terms of either tumour response or survival (Harvey *et al*, 1984; Taal *et al*, 1993; Okada *et al*, 1994). Conduct of large-scale clinical trials of chemotherapy for CBT is difficult because of its low incidence and the poor general condition of patients with CBT. Nevertheless, it is needed to develop new chemotheraputic regimens with better anticancer activity and better toxicity profile for patients with CBT.

Gemcitabine (2,2-difluorodeoxycytidine) is a novel antimetabolite active against lung, pancreas, breast, bladder and ovarian cancers (Kaye, 1994). Gemcitabine is currently the only drug approved by the FDA for the treatment of advanced pancreatic cancer (Burris *et al*, 1997). Since the biliary tract and pancreas share a common embryonal origin, the possibility that gemcitabine may also be active against CBT has recently been investigated. The results of these studies, most of them were small-series phase II trials, have generally supported a beneficial role of gemcitabine in the treatment of CBT (Raderer *et al*, 1999; Gebbia *et al*, 2001; Scheithauer 2002).

Weekly 24-h infusion of high-dose 5-FU (2600 mg m^−2^) and leucovorin (300 mg m^−2^), the HDFL regimen, has been demonstrated to be effective against colorectal and gastric cancers (Ardalan *et al*, 1991; Hsu *et al*, 1997; Yeh *et al*, 1997). Although the dose of 5-FU in HDFL is much higher than that of the conventional bolus 5-FU regimens such as the Mayo protocol, the resulting bone marrow toxicities are surprisingly low. For example, with HDFL alone, the likelihood of developing grade 3 or 4 haematological toxicities has been reported as below 3%. We have recently clarified the mechanisms responsible for the low marrow toxicities of the HDFL regimen (Yeh *et al*, 2000a). Further, in an *in vitro* experiment mimicking the pharmacokinetics of HDFL, prolonged exposure of gastric cancer cells to 2.5–5 *μ*M of 5-FU resulted in a more durable suppression of thymidylate synthase and enhanced cytotoxicity (Yeh *et al*, 2000b). The possibly better therapeutic index of HDFL in gastrointestinal tract malignancies, as compared with the bolus 5-FU regimens (O'Dwyer *et al*, 2001; Koehne *et al*, 2003, Saltz, 2003), has made it an ideal component for combination chemotherapy of CBT.

This study sought to clarify the effectiveness and toxicity of the combination of gemcitabine and HDFL in the treatment of CBT.

## PATIENTS AND METHODS

### Patients

Eligibility criteria for this study included (1) histologically or cytologically proven intrahepatic or extrahepatic cholangiocarcinoma, papilla vater carcinoma or gallbladder carcinoma that were inoperable because of either locally advanced disease (AJCC T4 classification, tumours invading main portal vein or hepatic artery or invading multiple extrahepatic organs or structures), or evidence of distant metastasis (AJCC M1 classification) (Fleming, 1997); (2) age between 18 and 70 years; (3) bidimensionally measurable disease; (4) Karnofsky performance status ⩾60%; (5) white blood cell (WBC) count ⩾4000 *μ*l^−1^, absolute neutrophil count ⩾1500 *μ*l^−1^, platelet count ⩾150 000 *μ*l^−1^, serum alanine and aspartate aminotransferases levels ⩽5 times upper normal limit, total bilirubin ⩽5 mg dl^−1^, serum creatinine ⩽1.5 mg dl^−1^ and serum triglyceride level ⩾70 mg dl^−1^. A low limit for serum triglyceride was set in order to avoid HDFL-related hyperammonemic encephalopathy (Yeh and Cheng, 1997). No prior cytotoxic chemotherapy was allowed, except for low-dose chemotherapy used as a radiosensitiser. Prior radiotherapy was acceptable if it had been completed at least 6 weeks before enrollment in this study and did not involve the index tumour lesion for evaluation of tumour response. This study was approved by the Institutional Review Board of National Taiwan University Hospital. All patients had signed informed consent prior to enrollment in the study.

### Treatment plan

The protocol treatment consisted of gemcitabine, 800 mg m^−2^ i.v. for 30 min, followed by 5-FU, 2000 mg m^−2^, plus leucovorin, 300 mg m^−2^, i.v. for 24 h, on days 1, 8 and 15. The treatment cycle was repeated every 4 weeks. The drugs were delivered via a Port-A catheter on an outpatient basis.

The doses of both gemcitabine and HDFL on days 8 and 15 within a cycle were reduced by 25% of the planned doses if the WBC count was less than 2500 *μ*l^−1^ or platelet count was less than 75000 *μ*l^−1^ on the scheduled day of administration, or if grade 3 nonhaematological toxicity (except for nausea/vomiting) occurred after the previous dose. Doses of gemcitabine and HDFL on days 8 and 15 were omitted if the WBC count was less than 1000 *μ*l^−1^ or platelet count was less than 50000 *μ*l^−1^, or if grade 4 nonhaematological toxicity (except for nausea/vomiting) occurred. A new cycle was started if the WBC count was more than 4000 *μ*l^−1^, platelet count was more than 100000 *μ*l^−1^, and nonhaematological toxicity (except for nausea/vomiting) was less than grade 3. If the patient did not recover from toxicity resulting from treatment on the scheduled day 1, the protocol treatment was postponed until the resolution of toxicity. If the new cycle had to be postponed for more than 8 weeks, the patient was removed from the protocol treatment.

### Evaluation of tumour response and toxicity

During the protocol treatment, the patients were evaluated every week with a routine history taking and physical examination. Hemogram was checked before each administration of protocol treatment, and serum biochemistry, electrolytes and prothrombin time were checked before each cycle. The tumour response was evaluated by imaging studies at least once every 2 cycles. Patients who received two or more cycles of the protocol treatment were considered evaluable for tumour response and those who had completed one or more cycles were considered evaluable for toxicity.

Tumour response and toxicity were evaluated according to World Health Organization criteria (Miller *et al*, 1981). Patients with progressive disease (PD) were removed from the protocol treatment. Patients with complete response (CR) received three additional cycles after the documentation of CR, and then the protocol treatment was stopped. Patients with partial response (PR) continued with the protocol treatment until PD or prohibitive toxicity developed. Patients with stable disease (SD) after four cycles of the protocol treatment could either continue with the protocol treatment until PD or prohibitive toxicity developed, or stop the protocol treatment at the discretion of the attending physician.

### Statistical analysis

Simon's optimal two-stage phase II trial design was used to estimate the number of patients needed in this study (Simon, 2001). The results of this estimation indicated that for a lower activity level of 5% and a higher activity level of 20%, at least one responders should be seen in the first 10 patients and a total of 29 patients should be accrued to obtain a false-positive rate of 5% and a false-negative rate of 10%.

The median follow-up time was calculated by constructing a Kaplan–Meier survival curve for all participating patients and reversing the ‘event’ and ‘censor’. The 50% point of this curve then indicated the median follow-up time (Shuster, 1991). Progression-free survival was defined as the duration from the date of starting the treatment to the date of documented disease progression, death by any cause, or last follow-up. Overall survival was defined as the duration from the date of starting protocol treatment to the date of patient death or last follow-up. Both the progression-free and the overall survival were calculated using the Kaplan–Meier method. The difference in clinical parameters between responders and nonresponders to protocol treatment was evaluated by χ^2^ test or Wilcoxon rank-sum test.

## RESULTS

### Patients

From April 1999 through November 2002, 30 patients (16 men, 14 women) were enrolled in this study. The median age was 55.2 years (range 30.8–72.5 years). The clinical characteristics of these patients are summarized in Table 1

**Table 1:** Clinicopathological features of the patients

**Parameters**	**Number of patients (%)**
*Karnofsky performance status* (%)	
100	2 (6.7)
90	13 (43.3)
80	10 (33.3)
70	3 (10.0)
60	2 (6.7)
	
*Primary site of tumour*	
Intrahepatic cholangiocarcinoma	16 (53.3)
Carcinoma of common bile duct	2 (6.7)
Carcinoma of ampulla vater	7 (23.3)
Carcinoma of gallbladder	5 (16.7)
	
*Prior therapies*	
Curative surgery	15 (50.0)
Palliative surgery	3 (10)
Radiotherapy	2 (6.7)
Chemoradiotherapy	1 (3.3)
TACE^a^	1 (3.3)
	
*Sites of metastases*	
Liver	25 (83.3)
Lung	5 (16.7)
Bone	1 (3.3)
Lymph nodes	8 (26.7)
Others^b^	5 (16.7)
	
*Biliary tract obstruction*	
Yes	14 (46.6)
No	16 (53.4)

a Transarterial chemoembolisation.

b Including adrenal gland, pleura, peritoneum, abdominal wall and duodenum.

. A total of 15 patients had recurrent disease after prior curative surgery and 15 patients had unresectable advanced or metastatic disease. A total of 14 patients had obstructive jaundice that required biliary drainage (13 percutaneous transhepatic biliary drainage; one internal biliary stent) before enrollment. At the end of periodic monitoring for this study on 30 September 2003, the median duration of follow-up was 43.6 months.

### Treatment

A total of 166 cycles of the protocol treatment were administered. The median number of cycles given per patient was 4 (range: 1–24). Dose or schedule modification was necessary in 10 patients. The causes of modification included infection (four patients), thrombocytopenia (three patients), leucopenia (two patients), anaemia (one patient) and hepatic toxicity (one patient).

### Response

In total, 28 patients were evaluable for response. No patient achieved CR, while six patients (four women; two men) achieved PR. The overall response rate was 21.4% (95% CI: 5.2–37.6%) for the evaluable patients and 20% (95% CI: 4.8–35.2%) for the intent-to-treat. There was no significant difference in the clinicopathological features between the responders and nonresponders in terms of age, sex, performance status, primary site of tumour (cholangiocarcinoma, gallbladder carcinoma, common bile duct carcinoma or ampula vater carcinoma), disease stage (locally advanced or metastatic) and pre-existing biliary tract obstruction. All responders had improvement in tumour-related symptoms and performance status. In all, 13 patients had SD and nine patients had PD. The performance status of all patients with SD remained stationary during the protocol treatment, with a median of four treatment cycles.

**Figure 1:**
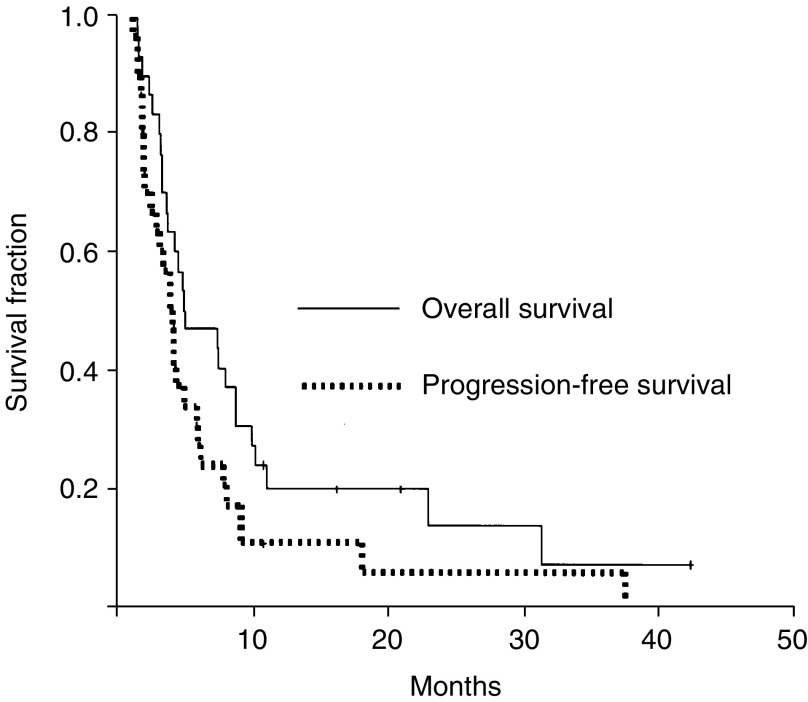
The overall and progression-free survival curves of the patients.

The median progression-free and overall survival for all of the 30 patients were 3.7 months (95% CI: 2.8–4.6 months) and 4.7 months (95% CI: 0.8–8.6 months), respectively (Figure 1

). At the end of periodic monitoring for this study, the overall survival for the six responders was 4.7, 10.2, 9.9, 23.4, 43.6+ and 10.8+ months, respectively.

### Toxicity

A total of 29 patients were evaluable for toxicity. As summarized in Table 2

**Table 2:** Toxicity profiles of gemcitabine–HDFL

	**WHO toxicity grades**			
	**1**	**2**	**3**	**4**
*Hematological*				
Leucopenia	9 (31.0)	3 (10.3)	2 (6.9)	2 (6.9)
Anaemia	8 (27.6)	14 (48.3)	2 (6.9)	1 (3.4)
Thrombocytopenia	1 (3.4)	3 (10.3)	0 (0.0)	3 (10.3)
				
*Non-haematological*				
Infection	0 (0.0)	5 (17.2)	5 (17.2)	4 (13.8)
Fever	3 (10.3)	16 (55.2)	1 (3.4)	0 (0.0)
Nausea/vomiting	12 (30)	3 (10.3)	2 (6.9)	0 (0.0)
Diarrhoea	8 (27.6)	5 (17.2)	0 (0.0)	1 (3.4)
Constipation	0 (0.0)	4 (13.8)	0 (0.0)	0 (0.0)
Alopecia	10 (33.3)	4 (13.3)	—	—
Hand–foot syndrome	1 (3.4)	0 (0.0)	0 (0.0)	0 (0.0)
Hepatic toxicity	2 (6.7)	4 (13.3)	0 (0.0)	3 (10.3)
Renal toxicity	3 (10.3)	1 (3.4)	1 (3.4)	1 (3.4)
Neurotoxicity	0 (0.0)	0 (0.0)	0 (0.0)	0 (0.0)

Values are number of patients (percentage).

, the most common grade 3 or 4 toxicity was infection, which occurred in nine patients. Eight of the nine patients had biliary tract infection and six of them had pre-existing biliary tract obstruction that required biliary drainage during the protocol treatment. The biliary tract infection resulted in withdrawal from the protocol treatment in five patients.

## DISCUSSION

In this study, we found that weekly gemcitabine plus HDFL was only moderately active for patients with advanced CBT. This regimen was in general well tolerated, but patients with underlying biliary tract obstruction were at increased risk of developing biliary tract infection.

The present study is one of the largest reported series of combination chemotherapy with gemcitabine for advanced CBT. The objective response rates of reported studies have varied widely and are difficult to compare because of possible bias in patient selection. In these previous studies, the dosage of gemcitabine ranged from 800 to 1200 mg m^−2^ week^−1^, but the optimal dosing and schedule of gemcitabine remained undetermined. Although a dose–response relationship for gemcitabine has been suggested by previous studies, the potential benefit of higher doses of gemcitabine must be balanced with the increased risk of toxicity. Although some investigators suggested that a fixed dose-rate infusion of gemcitabine may improve its antitumour activity, the clinical benefit of this approach remains to be determined. (Fossella *et al*, 1997; Touroutoglou *et al*, 1998; Tempero *et al*, 2003).

It has been suggested that addition of 5-FU/leucovorin will further improve the therapeutic efficacy of gemcitabine in CBT. Gebbia *et al* (2001) reported two consecutive studies of gemcitabine with or without 5-FU/leucovorin for patients with biliary tract cancer. They found that gemcitabine, 1000 mg m^−2^ i.v. on days 1, 8 and 15 every 5 weeks, had an objective response of 22% (95% CI: 6–48%) in 18 evaluable patients; while addition of 5-FU, 400 mg m^−2^ i.v. bolus, followed by 600 mg m^−2^ i.v. for 22 h, and leucovorin, 100 mg m^−2^ i.v. for 2 h, to gemcitabine, 1000 mg m^−2^, on days 1 and 8 every 3 weeks, produced an objective response of 36% (95% CI: 17–59%) in 22 evaluable patients. Addition of 5-FU/leucovorin did not appear to increase the severity of toxicity. The regimen of 5-FU/leucovorin used by Gebbia *et al*, first described by de Gramont *et al*, has been commonly applied in the treatment of metastatic colorectal cancer; and it has been suggested that higher total doses of 5-FU, similar to those used in our study, may further enhance the antitumour activity of 5-FU/leucovorin (de Gramont *et al*, 1998; Koehne *et al*, 1998). Further studies are warranted to explore the optimal dosing and combination schedule for gemcitabine and 5-FU/leucovorin infusion.

Preliminary reports of other gemcitabine-containing chemotherapy regimens have shown promising activity for patients with advanced CBT. Objective response rate of more than 30% for patients with advanced CBT was reported by using the combinations of gemcitabine with cisplatin or oxaliplatin (Andre *et al*, 2001; Carraro *et al*, 2001; Doval *et al*, 2001). It is difficult, however, to compare directly the response rate in different trials because of the relatively small sample size and the heterogeneous patient populations of their series. In these and also the report by Gebbia *et al*, gallbladder cancer comprised the majority of the patient population. In contrast, intrahepatic cholangiocarcinoma was the most common diagnosis in our study. The relationship between chemosensitivity and cellular origin of CBT, as well as the differential activity of gemcitabine, cisplatin and oxaliplatin on advanced CBT, remains to be clarified.

The relatively high incidence of biliary tract infection in our patients is an important concern. Concurrent grade 3 or 4 myelotoxicity was rare in our study, and pre-existing obstructive jaundice and the relatively poor general condition were the most important risk factors for developing biliary tract infection. The relatively poor general condition of our patients was also suggested by the short median overall survival (4.7 months), which compared unfavourably with those reported in other series (5–12 months) (Hejna *et al*, 1998; Gebbia *et al*, 2001). Therefore, careful selection of patients for chemotherapy is essential to decrease the incidence of this complication. On the other hand, cumulative toxicity resulting from the gemcitabine plus HDFL regimen was infrequent. Two of our patients had received more than 10 cycles of protocol treatment and remained in PR without evidence of any cumulative toxicities at the end of periodic monitoring for this study.

We conclude that gemcitabine plus HDFL is well tolerated and modestly active in selected patients with advanced CBT. Improvement in the quality of life can be reasonably expected in patients who respond to chemotherapy.
